# Unwind to the beat: chromatin and cardiac conduction

**DOI:** 10.1172/JCI165663

**Published:** 2023-02-01

**Authors:** Douglas J. Chapski, Thomas M. Vondriska

**Affiliations:** 1 Department of Anesthesiology and Perioperative Medicine,; 2 Department of Medicine, and; 3 Department of Physiology, David Geffen School of Medicine at UCLA, Los Angeles, California, USA.

## Abstract

How chromatin accessibility and structure endow highly specialized cells with their unique phenotypes is an area of intense investigation. In the mammalian heart, an exclusive subset of cardiac cells comprise the conduction system. Many molecular components of this system are well studied and genetic variation in some of the components induces abnormal cardiac conduction. However, genetic risk for cardiac arrhythmias in human populations also occurs in noncoding regions. A study by Bhattacharyya, Kollipara, et al. in this issue of the *JCI* examines how chromatin accessibility and structure may explain the mechanisms by which noncoding variants increase susceptibility to cardiac arrhythmias. We discuss the implications of these findings for cell type–specific gene regulation and highlight potential therapeutic strategies to engineer locus-specific epigenomic remodeling in vivo.

## Noncoding regions and the risk for cardiac arrhythmias

Proper electrophysiological signaling through the cardiac conduction system is necessary for postnatal life. Thus, genetic variation that completely disrupts cardiac conduction in early life is only rarely observed. More common is a heritable predisposition to cardiac arrhythmias, often coincident with cardiomyopathy, wherein the genetic code establishes a cardiac substrate more vulnerable to improper conduction. This vulnerable substrate can manifest in ventricular or atrial myocytes, as a tissue-level phenomenon of the cardiac conduction system itself, or through the interactions of specialized cells of the heart with the vasculature, the immune system, and the myriad of environmental factors that trigger cardiac arrhythmias.

**Figure 1:**
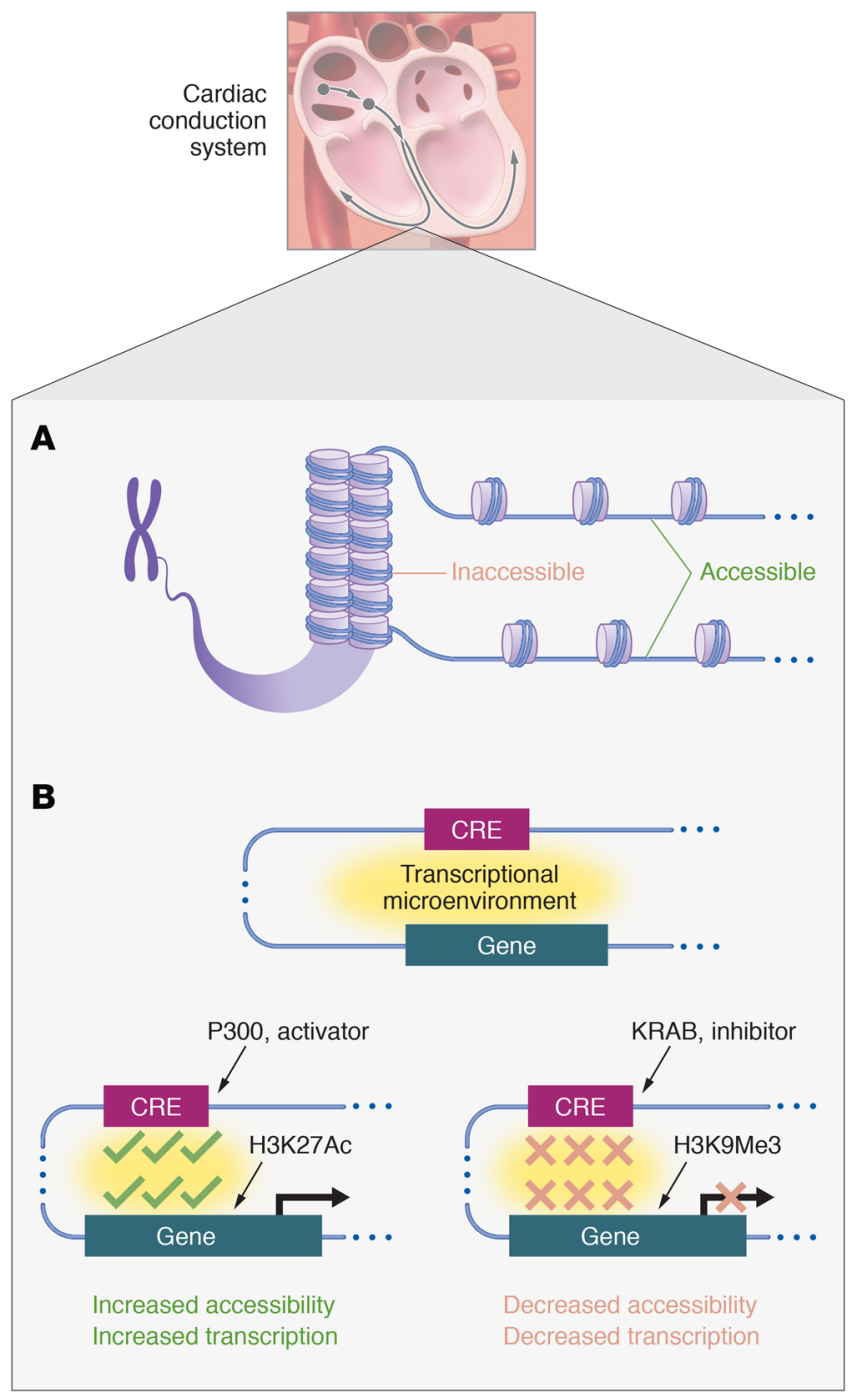
Chromatin accessibility may be targeted for gene regulation of the cardiac conduction system in vivo. (**A**) Cells of the cardiac conduction system possess specific chromatin accessibility regions (3). Nucleosome density determines local chromatin accessibility, with less dense nucleosome packing allowing for gene access and transcription. (**B**) Cardiac conduction system cells also possess CRE (3). Transcriptional microenvironments are created when CRE (i.e., distal enhancers) come into contact with genes. In vivo strategies to alter chromatin accessibility and transcriptional microenvironments could alter transcription in cells specific to the CCS. For example, epigenetic targeting using a dCas9-dependent experimental design could activate transcription via P300-mediated acetylation of histone H3K27 or inhibit transcription via KRAB-mediated histone methylation. Such treatments may modify heart rhythm phenotypes.

Genetic variation associated with cardiac conduction system (CCS) diseases is only partially understood, with a proportion of this variation mapping in or near ion channels, transcription factors, or other proteins (1). GWAS reveal arrhythmia risk-conferring variation in nonprotein coding regions of the genome (2). The question of how to interpret this heritable risk for arrhythmias thus becomes a question of understanding epigenetic regulation of the various specialized cells of the heart. In this endeavor, it can be helpful to consider two properties of chromatin that are related but distinct: (a) global chromatin architecture, or how the linear chromosomes are packaged within the cell’s nucleus, and (b) local chromatin accessibility, or how the positioning of nucleosomes and other chromatin structural proteins at a given protein-coding gene or regulatory element render it suitable for transcription (Figure 1A). The premise: noncoding SNPs may exert disease-causing effects by altering accessibility of regulatory elements such that the tortuous configuration of the genome in a living cell positions these noncoding SNPs in close apposition with genes that are directly involved in disease. In the context of a study in this issue of the *JCI*, Bhattacharyya, Kollipara, et al. examined SNPs in noncoding genomic regions that associate with risk for cardiac arrhythmias (3). The authors investigated whether the mechanisms by which this genetic variation causes disease might involve perturbation of chromatin accessibility and structure (3).

More compact genomic regions reflect a chromatin landscape where genes are typically repressed, while more accessible chromatin is associated with active transcription. These regions can be distinguished across the genome using the assay for transposase accessible chromatin followed by short read sequencing (ATAC-Seq) (4). ATAC-Seq distinguishes accessible from inaccessible chromatin and was employed by Bhattacharyya, Kollipara, et al. to examine lineage-specific accessibility in the CCS, including in the specialized cells of the sinoatrial node (SAN), the atrioventricular node (AVN), and the ventricular conduction system (VCS) comprising the bundle of His and Purkinjie fibers. Previous studies have examined accessibility and structure in the whole heart and in myocytes (5), but a key feature of the epigenome is its cell-type specificity. Driven by the actions of lineage-specific transcription factors, chromatin modifying proteins, and other developmental cues, cells of the body adopt distinct chromatin-accessibility patterns and structures that enable and restrict the phenotypes the cell may adopt. To explore this phenomenon in the context of the CCS, the authors leveraged a custom isolation of nuclei tagged in specific cell types (INTACT) approach to purify nuclei from transgenic mice. To explore this phenomenon in the context of the CCS, the authors leveraged a custom isolation of nuclei tagged in specific cell types (INTACT) approach to purify nuclei from transgenic mice whose Sun1-sfGFP-myc locus is preceded by a loxP-STOP-loxP cassette, crossed with Cre lines driven by Shox2 (6), Gjd3 (7), or Cntn2 (8) promoters, which are markers for SAN (9), AVN (10), and VCS cells (11) respectively. Using isolated CCS cell-type specific nuclei (marked by Sun1-GFP), the authors interrogated the local chromatin accessibility landscape by performing ATAC-Seq. This approach identified ATAC peaks present in different cell types (11 peaks in SAN, 12 in AVN, and 10 in VCS) that were not observed in the available ENCODE data sets from the whole heart, highlighting the utility of examining nuclei in a cell type-specific manner (3).

The authors then examined these regulatory elements for putative transcription factor motifs, finding EWSR1-FLI1 and ONECUT1 to be potential modifiers of these regulatory elements. Because the EWSR1-FLI1 fusion protein is cancer specific and thus is not expressed in healthy myocytes, the authors overexpressed the transcription factor Etv1. Etv1 and EWSR1-FLI1 are both ETS-family transcription factors. These factors or ONECUT1 were expressed in separate neonatal rat ventricular myocyte experiments to show that these factors alone induce transcription of several known target genes. The authors also performed chromatin immunoprecipitation followed by quantitative PCR to show direct binding of EWSR1-FLI1 and ETV1 to *Myh6* and *Actb* target loci, as well as binding of ONECUT1 to the promoters of 5 target genes, suggesting that these transcription factors may interact with regulatory elements in addition to the target genes themselves (3).

## Cell type–specific enhancer regions

Enhancers exist in CCS, but are they specific to CCS cell types? Next, the authors used their ATAC-Seq data to identify CCS cell type-specific enhancer regions, which are thought to promote expression of nearby genes. Interestingly, the authors found 27 CCS-specific enhancers when comparing their ATAC-Seq data set to a cardiomyocyte ATAC-Seq data set and the VISTA database (12). Fixed embryos were available for 22 of these enhancers from VISTA, 14 of which were functionally validated in the CCS using lacZ tracing in mouse embryos. The authors conducted a more detailed analysis on three of these enhancers in primary CCS cells: one in an intron near *Btbd9* (SAN), a second in an intergenic region between *Rhob* and *Hs1bp3* (AVN), and a third in an intron of *Igf1r* (VCS) (3).

How do these enhancers function? Analysis of SNPs identified from GWAS of cardiac rhythm disorders revealed that CCS enhancers were enriched 12-fold for these SNPs compared with cardiomyocyte enhancers (3). This observation highlights the utility of regulatory element identification in specialized cell types and implies that the SNP may exert its effects on gene expression and phenotype by disturbing the enhancer. The authors then performed a detailed study of intronic or intergenic SNPs in accessible chromatin regions associated with heart rate, QRS interval, Q-T interval, and PR interval, again validating them with luciferase assays in primary cells. Moreover, for a PR-interval SNP overlapping a SCRT1/2 predicted binding site, the authors demonstrate that SCRT1 bound to a regulatory element within an intron of *Cav1* — and that a variant allele in this regulatory region was sufficient to attenuate transcription in a luciferase assay (3).

Genomic regulatory elements were originally thought to regulate their closest genes, however, high-throughput chromatin conformation capture studies have revealed that such regions can directly interact with distal genes (13). To provide a chromatin architectural context for the actions of these regulatory elements, Bhattacharyya, Kollipara, and colleagues used a published promoter capture Hi-C data set (14). This method allowed the authors to explore chromatin contacts surrounding putative regulatory elements and their hypothesized target genes measured in human induced pluripotent stem cell–derived cardiomyocytes. In other words, Bhattacharyya, Kollipara et al. provide evidence that the structure of the genome may be specifically tuned to bring regulatory elements into close apposition with genes involved in heart rhythm phenotypes, such that disease-causing variation in a regulatory element may act by disrupting expression of the target genes. These observations are important because they provide the basis to test the hypothesis that genetic variation in noncoding regions acts through features endowed by chromatin structure. The overall findings of the paper are also notable because they provide a glimpse into CCS-specific regulatory elements—a cell type-specific epigenome annotation effort that will enable future studies of CCS gene regulation.

## Conclusion and future directions

Future efforts are needed to develop a comprehensive map of CCS-specific chromatin structure, since promoter-capture Hi-C only enriches for chromatin interactions with known promoters. Furthermore, whether the phenotypes and genome regulatory interactions in vivo are recapitulated in induced pluripotent stem cell–derived cardiomyocytes is unknown. To determine whether the interactions shown in the manuscript exist in murine CCS cells, single-cell Hi-C experiments optimized for very low input cell numbers could be performed on isolated SAN, AVN, and VCS cells. This strategy would reveal, with single-cell granularity, whether the identified regulatory regions from Bhattacharyya, Kollipara, et al. directly interact with their target genes in three dimensions.

If these molecular details of chromatin interactions get us closer to targeting noncoding regions therapeutically, how would such targeting proceed? One approach would involve changing the transcriptional and regulatory landscape of the enhancer region. Tools have recently emerged to enable temporally regulated, reversible modification of gene expression from the endogenous locus (15). For activation, overexpression of an enzymatically dead Cas9 (dCas9) protein fused to the histone acetyltransferase P300 — which deposits acetyl groups on histone H3 lysine 27, promoting gene expression — with a guide RNA targeting a specific distal regulatory element would determine whether making that element more accessible could induce gene transcription (Figure 1B). The converse experiment would indicate whether heterochromatin formation can be induced directly within a regulatory element. Here, overexpression of Krüppel-associated box (KRAB) fused to dCas9 to recruit histone H3 lysine 9 methylation machinery would reveal whether rendering a regulatory element less accessible could reduce transcriptional output at a distal gene. Performing such experiments in vivo might be feasible given recent advances in adeno-associated virus 9 based transcriptional modulation of cardiomyocytes in mice (16). Such experiments would definitively test the role of regulatory region accessibility in distal gene transcription in vivo.

Another approach could be the administration of small molecules that modulate epigenetic factors, such as inhibitors of histone deacetylases and BET/bromodomain proteins, which are well tolerated in vivo and have been shown to ameliorate various cardiac pathologies (e.g., hypertrophy and fibrosis) (17) but have never been tested in arrhythmias. In principle, these compounds could work by modulating histone modifications or histone binding proteins at regulatory elements, such as those identified in Bhattacharyya, Kollipara, et al. (3), changing accessibility and perhaps chromatin structure, albeit using a nonlocus specific strategy.

Subpopulations of CCS cell types revealed from single-cell experiments could inform disease mechanisms, and coupled with epigenomic measurements like those in Bhattacharyya, Kollipara, et al., could reveal how gene expression is controlled in these specialized cells. Because CCS cells are difficult to visualize during cardiac procedures, putative markers could reveal specific subpopulations of the SAN, AVN, and VCS, perhaps using a recent conjugated antibody approach developed to mark CCS cell–surface markers (18). Such surface markers combined with single cell epigenomic measurements may shed light on the fundamental mechanisms driving CCS function, as well as on general questions of gene regulation in scarce, peculiar cells.

## Version 1 02/01/2023

Electronic publication
